# School‐based surveillance for influenza vaccine effectiveness during 2014‐2015 seasons in Hong Kong

**DOI:** 10.1111/irv.12455

**Published:** 2017-05-25

**Authors:** Ting F. Leung, Renee W. Y. Chan, Angela Kwok, Wendy C. S. Ho, Mars K. P. Tao, Kam L. Hon, Frankie W. T. Cheng, Albert M. Li, Paul K. S. Chan

**Affiliations:** ^1^ Department of PaediatricsPrince of Wales HospitalThe Chinese University of Hong KongShatinHong Kong; ^2^ Department of MicrobiologyPrince of Wales HospitalThe Chinese University of Hong KongShatinHong Kong

**Keywords:** epidemiology, influenza, surveillance, vaccine effectiveness

## Abstract

**Background:**

Influenza imposes substantial healthcare burden in children, which can be prevented by vaccination. Influenza vaccination coverage varies widely among childhood populations worldwide, which has significant impact on herd immunity and usefulness of influenza vaccine. However, there are limited real‐life data on influenza vaccine effectiveness (VE) in children.

**Objective:**

This prospective study aimed to investigate clinical spectrum of childhood influenza and VE in preventing influenza in Hong Kong children.

**Methods:**

A total of 623 children were recruited from 15 kindergartens and primary schools. Parents completed a questionnaire on subjects’ health and influenza vaccination history. Flocked nasopharyngeal swabs (FNPSs) were collected in biweekly school visits during 2014‐2015 influenza seasons. Influenza A and B viruses were detected and typed by molecular assays.

**Results:**

A total of 2633 FNPS samples were collected, with two or more samples being obtained from 607 (97.4%) of subjects. Thirty‐six (11.2%) subjects had influenza A or B in 2014, whereas all 19 (6.3%) subjects identified in 2015 had influenza A. Ninety‐nine subjects reported influenza‐like illness (ILI), and nine illness visits were arranged. Influenza vaccination was protective against ILI but not mild laboratory‐confirmed influenza by surveillance. Moderate overall influenza VE of 42%‐52% was observed for ILI, and subgroup analyses showed much higher VE for both ILI (70.9% vs 34.6%) and mild laboratory‐confirmed influenza (44.0% vs −6.2%) in school‐age children than preschoolers who were vaccinated within 12 months.

**Conclusions:**

Mild laboratory‐confirmed influenza infection is common in children during influenza seasons. Influenza vaccination is effective against ILI but not mild infection identified by surveillance.

## 1 INTRODUCTION

Influenza is an important healthcare burden in Hong Kong^1^, ^2^, and influenza vaccination reduced laboratory‐confirmed influenza by 59% and influenza‐like illness (ILI) by 36% in healthy children.^3^ However, there are limited post‐licensure effectiveness data in Asian children. In the European sentinel surveillance networks, seasonal influenza vaccine had low‐to‐moderate effectiveness of 43% against influenza A (H3) in the early 2011/2012 season.^4^ Similar level of vaccine effectiveness (VE) was reported from 19 influenza surveillance sites in Guangzhou^5^, supporting the need to delineate the effectiveness of seasonal influenza vaccine in children. The reported figure of laboratory‐confirmed cases underestimates true case numbers as surveillance only focused on severe cases. More than one‐third of household contacts of A(H1N1)pdm09‐infected patients who had serologic evidence of pandemic influenza were asymptomatic.^6^ Children with mild influenza infection impose a public health concern by perpetuating influenza transmission in the community. Nonetheless, there is a lack of studies that reported influenza VE in preventing asymptomatic or mildly symptomatic influenza. Our smaller local study with biweekly nasopharyngeal sampling revealed that influenza B virus was detected in 18% and 30% of asymptomatic and sick children.^7^ Influenza surveillance in prospective cohorts is thus necessary to define the full spectrum of influenza. Because of viral antigenic drift, annual estimates of influenza VE would provide important data to monitor any change in the impact of seasonal influenza vaccination program.^8^ This point was illustrated by the surge of influenza in 2014/2015 season due to mismatch between vaccine A/H3N2 strain and the new circulating A/H3N2_Switzerland strain (http://www.chp.gov.hk/en/view_content/18632.html).^9^ The Centre for Health Protection (CHP) of Hong Kong recommended influenza vaccine containing A/California/7/2009 A(H1N1)pdm09‐like virus, A/Switzerland/9715293/2013 (H3N2)‐like virus, and B/Phuket/3073/2013‐like virus for the 2015/16 season. This school‐based surveillance study identified the spectrum of both subclinical and clinical influenza in children and risk factors for such infections in Hong Kong preschool and school‐age children. We also aimed to report influenza VE against influenza and ILI in these two pediatric age groups.

## 2 MATERIALS AND METHODS

### 2.1 Study population

This prospective cohort study recruited children in two grades of kindergartens (K.1 [~3 years old] and K.3 [~5 years old]) and primary schools (P.2 [~7 years old] and P.5 [~10 years old]) in the Kowloon and New Territories Easter regions of Hong Kong that have a residing population of 300 000 children aged below 15 years according to census data in 2011. For our sampling strategy, our team generated the first randomly selected batch of 20 kindergartens and 20 primary schools from all those registered with the Education Bureau of Hong Kong Government, contacted the school principals for participation in this surveillance study, and then generated additional random batches until our target sample size of 560 children was achieved. We recruited about half of target sample size from all eligible classes of kindergartens and another half from all eligible classes of primary schools. At the study start, we contacted principals of selected kindergartens and primary schools to obtain their permission to join this study. Parents of eligible students in these schools then gave informed written consent for their children to participate in this surveillance. This study did not set any exclusion criterion for subjects in an attempt to recruit a representative study population. Based on local data^3^, we targeted this surveillance in consecutive influenza seasons in 2014‐2015. The Joint Chinese University of Hong Kong‐New Territories East Cluster Clinical Research Ethics Committee approved this study.

### 2.2 Subject assessment

Parents completed a questionnaire that recorded subjects’ demographics, pre‐existing medical illnesses, and influenza vaccination history within 3 years. Our staff verified vaccination history against immunization cards or with responsible doctors. Subjects with the following criteria were considered vaccinated: (i) ≥14 days post‐vaccination; (ii) received two doses 28 days apart if vaccinated for the first time; or (iii) received at least one dose in a previous influenza season and one dose in the season under study.^5^ For surveillance samples, serial flocked nasopharyngeal swabs (FNPSs) were collected every 2 weeks during school visits regardless of whether subjects had respiratory symptoms. *Surveillance* samples were collected between February and June in 2014 and between January and February in 2015. We started school surveillance within 2 weeks upon announcement of the start of influenza seasons by our CHP. This active influenza surveillance could detect children with asymptomatic or mildly symptomatic infections, which provided an unbiased data on the full spectrum of influenza infections.

Our staff phoned families every 2 weeks to remind subjects about the next surveillance visits and enquire whether they had ILI defined based on modified World Health Organization (WHO) case definition (ie, fever ≥38°C *plus* two of the followings: cough, sore throat, rhinorrhea, myalgia, headache)^10^ and whether they recently received influenza vaccination. Parents were provided our contact phone number and encouraged to inform us as soon as their children developed ILI. Our team attempted to arrange illness visit for all subjects with ILI during influenza seasons in early 2014 and 2015, and those agreed to attend illness visit returned to our outpatient clinic within 48 hours of ILI onset. During these visits, our nurse collected *illness* sample and recorded clinical features and concurrent medications.

### 2.3 NPS collection and processing

Nasopharyngeal sample was collected using flocked swab (Copan Diagnostics, Corona, CA).^11^, ^12^ Swabs taken from both nostrils of a subject were placed in the same specimen bottle containing viral transport medium and transported within 4 hours at room temperature to virology laboratory, where swabs were discarded after vortexing for 20 seconds to release the cells. Viral transport medium was then centrifuged and the pellet resuspended in 1 mL buffered saline and stored at −80°C until analyses.

### 2.4 Real‐time PCR for influenza virus detection

Viral RNA was extracted with PureLink^®^ Viral RNA/DNA Mini Kit (Life Technologies, Waltham, MA, USA) according to manufacturer's instructions. RNA was eluted with 50 μL sterile RNAse‐free water and stored at −80°C before use. Influenza A and B M gene‐specific real‐time (RT)‐PCR was performed in parallel using SuperScript^®^ III Platinum^®^ One‐Step Quantitative RT‐PCR System with ROX (Life Technologies). The RT‐qPCR reaction contained 5 μL of purified RNA, 0.5 μL of SuperScript^®^ III/Platinum^®^ T*aq* Mix (Life Technologies), 12.5 μL of 2× reaction mix in a final reaction volume of 25 μL. The sequences of primers for influenza A were as follows: (FLUAM‐7F) CTT CTA ACC GAG GTC GAA ACG TA; (FLUAM‐161R) GGT GAC AGG ATT GGT CTT GTC TTT A; and (FLUAM‐49‐P4) YAK‐TCA GGC CCC CTC AAA GCC GAG‐BHQ1. The primers for influenza B included the following: (FLUBHA‐108F) AGG GGA GGT CAA TGT GAC TG; (FLUBHA‐209R) GGG CAT AGT TTC CCT CTG GT; and (FLUBHA‐165P) FAM‐TTT TGC AAA TCT CAA AGG A‐MGB. The concentrations were 0.3 μMol L^−1^ for influenza A primers and 0.24 μMol L^−1^ and 0.2 μMol L^−1^ for influenza B primers. The cycling conditions were 50°C for 30 minutes, 95°C for 2 minutes, followed by 40 cycles of 95°C for 15 seconds and 60°C or 56°C for 30 seconds, for influenza A or influenza B respectively. RNA standards were prepared using the MEGAscript^®^ T7 Transcription Kit (Life Technologies), aliquoted, and stored at −80°C. To generate the standard curve, 10‐fold serial dilutions of the RNA transcripts were performed to cover the concentration range of 10^6^‐10^1^ copies/μL of standard. Amplification, detection, and data analysis were performed with StepOnePlus^™^ Real‐Time PCR System (Life Technologies).

### 2.5 Typing of influenza A and B viruses

The molecular diagnosis of influenza virus from 28 influenza A positive and 31 influenza B positive samples were based on the WHO guidelines (http://www.who.int/csr/disease/influenza/manual_diagnosis_surveillance_influenza/en/index.html) with slight in‐house modifications. Extracted RNA (50 ng) was subjected to reverse transcription using Superscript II (Life Technologies). For the subtyping of the influenza, primers were based on WHO guidelines which targeted the hemagglutinin gene‐specific for A(H1N1)pdm09, A/H3, B/Victoria‐lineage, and B/Yamagata‐lineage. Conventional PCR were performed using AmpliTaq Gold 360 Master Mix (Life Technologies), and the PCR products were resolved in 2% agarose gel. Samples showing expected band size after 50 amplification cycles were considered positive.

Viral load was expressed in the absolute copy numbers of influenza M gene determined from the standard curves generated from a standard plasmid with a known copy number in serial dilutions, which was included in the quantitative PCR simultaneously, as previously described.^13^ Real‐time PCR was conducted using SYBR Premix EX Taq master mix (Takara Kusatsu, Shiga, Japan), and the results were analyzed using PRISM 7900HT system (Applied Biosystems, Foster City, CA, USA).

### 2.6 Statistical analysis

Numerical data were expressed either in mean and standard deviation (SD) or median and interquartile range (IQR) as appropriate. The occurrence of laboratory‐confirmed influenza or ILI in relation to vaccination was analyzed by logistic regression, adjusting for covariates including seasonality (month of study), subjects’ age, sex, body mass index, and comorbid medical conditions. Surveillance data from all influenza seasons were combined during such analyses. Influenza VE was estimated by the “test‐negative case‐control” design according to published method.^14^ As vaccine recipients may have a greater likelihood of seeking health care should they develop infections, this analytical approach adjusts implicitly for this confounding which would otherwise bias VE. All analyses were performed two‐tailed using SPSS v.21 (Chicago, IL, USA), with 0.05 being the level of significance.

## 3 RESULTS

### 3.1 Study population

Five of 97 invited primary schools and 10 of 238 invited kindergartens agreed to participate. A total of 630 children aged 7.3±2.4 years were recruited, including 322 children in 2014 and 308 children in 2015. All subjects participated either in 2014 or 2015 seasons but not both. Seven subjects (one in 2014 and six in 2015) withdrew consent before FNPS collection. A total of 333 (53.5%) subjects received influenza vaccination within 3 years, with 78, 75, and 180 children being vaccinated in one, two, and 3 years, respectively.

**Figure 1:**
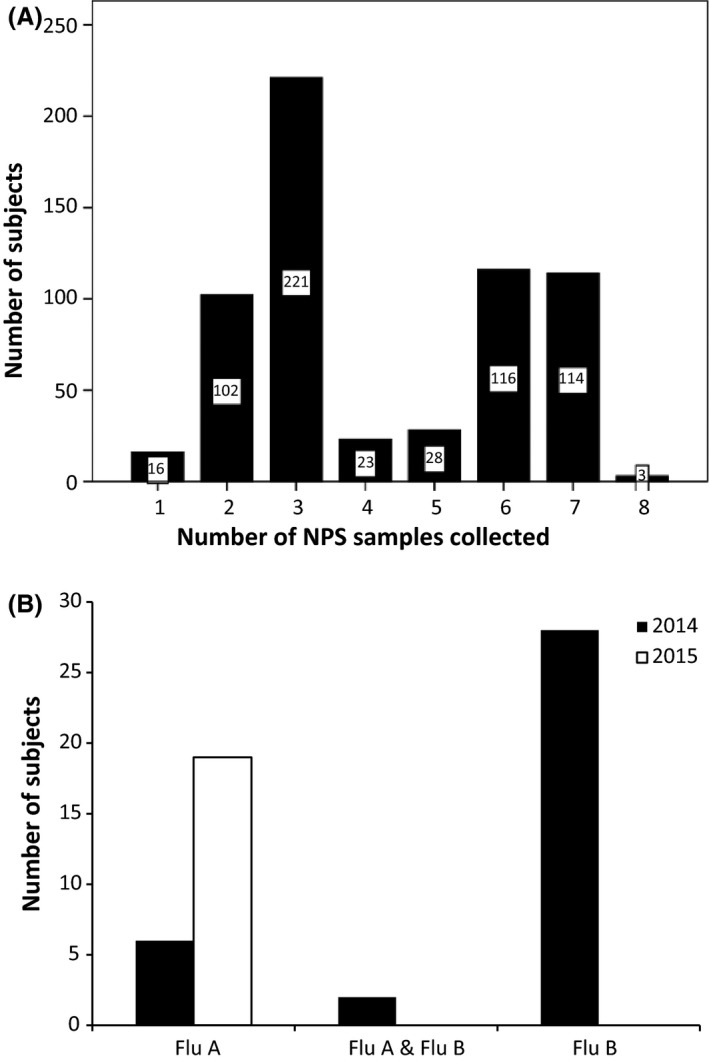
(A) Distribution of FNPS sample collection from our subjects, with two or more FNPS being collected from 320 (99.4%) subjects in 2014 and 295 (95.8%) subjects in 2015; and (B) detection of influenza A and B viruses by surveillance in years 2014 and 2015

Table 1 summarizes the characteristics of children who provided ≥one surveillance sample. Most children recruited in 2014 provided six to seven samples and those recruited in 2015 gave two to three samples (Figure 1A). A total of 2633 FNPS samples were collected from 623 children. Two FNPSs were obtained from 607 (97.4%) subjects and three samples from 505 (81.1%) subjects. ILI episodes were reported by 99 subjects, consisting of 63 (26.7%) of 236 preschoolers and 36 (9.3%) of 387 school‐age children (*P*<.001). FNPS was not obtained from these subjects because of unavailability of research staff to conduct home visit. In addition, nine illness visits were arranged for five subjects. There was no ILI outbreak in schools or reported transmission of influenza within the same classes and household of influenza‐infected children.

**Table 1:** Demographic and clinical features of study participants in 2014 and 2015

Parameter	Result	
	2014 (n=321)	2015 (n=302)
Age in years, mean±SD	7.0±2.7^a^	7.7±2.1
Male	173 (53.9)	162 (53.6)
Born in Hong Kong	257 (80.1)	260 (86.1)
Paternal secondary or university education	243 (75.7)	232 (76.8)
Maternal secondary or university education	248 (77.3)	240 (79.5)
History of influenza vaccination		
Within 3 y	162 (50.5)	171 (56.6)
At 25‐36 mo ago	115 (35.8)	125 (41.4)
At 13‐24 mo ago	127 (39.6)	129 (42.7)
Within 12 mo	134 (41.7)	132 (43.7)
History of pneumococcal vaccination	148 (46.1)	123 (40.7)
Asthma phenotypes		
Wheeze ever	59 (18.4)	49 (16.2)
Current wheeze	40 (12.5)	26 (8.6)
Asthma ever	21 (6.5)	18 (6.0)
Rhinitis ever	103 (32.1)	119 (39.4)
Eczema ever	81 (25.2)	84 (27.8)
Environmental exposures		
Breastfeeding ever	187 (58.3)	160 (53.0)
Breastfeeding for 4 mo and longer	106 (33.0)	87 (28.8)
Current cat/dog keeping	25 (7.8)	28 (9.3)
Current bird keeping	3 (0.9)	1 (0.3)
Current maternal smoking	28 (8.7)	29 (9.6)
Maternal smoking during infancy	11 (3.4)	5 (1.7)
Maternal smoking during pregnancy	9 (2.8)^b^	1 (0.3)
Current domestic smoking exposure	137 (42.7)	114 (37.7)
Indoor dampness or visible molds	122 (38.0)	101 (33.4)

Results expressed in number (percentage) unless stated otherwise.

a *P*<.001.

b *P*<.05.

### 3.2 Detection of influenza virus in surveillance samples

Influenza A and B viruses were detected in surveillance samples from 27 to 30 subjects respectively, with median (IQR) viral loads of 918 (99‐14 864) copies/μL and 262 (98‐324 027) copies/μL, and which were similar between preschool and school‐age children (*P*>.15). Figure 1B illustrates influenza detection in 2014 and 2015. Influenza B predominated in 2014 and influenza A in 2015 (*P*<.001). One subject had influenza A and B co‐infection in February 2014, and another subject had influenza A in February 2014 followed by influenza B 4 weeks later. Overall, 36 (11.2%) of 321 subjects had influenza A or B infection in 2014 whereas all 19 (6.3%) of 302 subjects had influenza A infection in 2015.

Six influenza A and 11 influenza B were not typable due to low virus load. For the remaining isolates, seven influenza A were A(H1N1)pdm09 and 14 were H3‐positive whereas among influenza B isolates, all detected in 2014, nine were in Yamagata‐lineage and 10 belonged to Victoria‐lineage. Influenza A (total seven) was typed into four A(H1N1)pdm09 and one A/H3 in 2014 and three A(H1N1)pdm09 and 13 A/H3 in 2015 (*P*=.025). None of these children were reported by parents to be “sick” at the time of FNPS collection. Besides, our nurses did not notice significant respiratory symptoms in these children at the time of FNPS collection. All illness FNPS samples were negative for both influenza A and B.

### 3.3 Relationship between ILI and subjects’ demographic and clinical factors

Table 2 summarizes the relationship between occurrence of ILI and subjects’ personal, clinical, and vaccine factors. Subjects with ILI were younger than those without ILI (*P*<.001), but ILI was not associated with any environmental or clinical factor. Influenza vaccination at all time points was protective against ILI (*P*=.002‐.022). Logistic regression confirmed such association for influenza vaccination within 3 years (odds ratio 0.49, 95% confidence interval 0.29‐0.81; *P*=.005). None of the other factors enlisted in Table 2 was associated with mild laboratory‐confirmed influenza detected by surveillance (data not shown).

**Table 2:** Relationship between occurrence of ILI and demographic, environmental, and allergy factors

Factor	ILI (n=99)	No ILI (n=524)	*P* a
Male	55 (55.6)	272 (51.9)	.505
Age in years, mean±SD	5.8±2.2	7.6±2.4	<.001
Born in Hong Kong	87 (87.9)	423 (80.7)	.090
Environmental exposures			
Breastfeeding ever	60 (60.6)	282 (53.8)	.213
Current domestic smoking exposure	39 (39.4)	210 (40.1)	.899
Current maternal smoking	8 (8.1)	48 (9.2)	.731
Current dog/cat keeping	10 (10.1)	43 (8.2)	.535
Indoor dampness or visible molds	41 (41.1)	180 (34.4)	.178
Presence of elder brother	24 (24.2)	117 (22.3)	.676
Presence of elder sister	25 (25.3)	103 (19.7)	.206
Allergy phenotypes			
Wheeze ever	18 (18.2)	89 (17.0)	.772
Current wheeze	12 (12.1)	53 (10.1)	.549
Asthma ever	5 (5.1)	34 (6.5)	.588
Use of asthma medication in past 12 mo	4 (4.0)	13 (2.5)	.329
Rhinitis ever	31 (31.3)	188 (35.9)	.383
Eczema ever	26 (26.3)	137 (26.1)	.981
History of influenza vaccination			
Within 3 y	39 (39.4)	294 (56.1)	.002
At 25‐36 mo ago	28 (28.3)	212 (40.5)	.022
At 13‐24 mo ago	27 (27.3)	229 (43.7)	.002
Within 12 mo	29 (29.3)	237 (45.2)	.003
Ever received pneumococcal vaccine	50 (50.5)	220 (42.0)	.117

ILI, influenza‐like illness; SD, standard deviation.

Results expressed in number (percentage) unless stated otherwise.

a Analyzed by Student's *t* test for age and χ^2^ or Fisher's exact test for other variables as appropriate.

### 3.4 Effectiveness of influenza vaccination

Sixty‐three (18.9%) subjects who received influenza vaccination within 3 years had either IL‐I or laboratory‐confirmed influenza detected by surveillance, which was significantly lower when compared to 86 (29.7%) children who were not vaccinated (*P*<.005). Table 3 illustrates the results for influenza VE in our children. Influenza vaccination was protective against ILI (*P*<.005) but not mild laboratory‐confirmed influenza when subjects were vaccinated within 3 years or 12 months. Influenza vaccine was moderately protective against ILI with VE varied between 42.1% and 51.9% when subjects were vaccinated at different time points before this study. On the other hand, influenza vaccination could not prevent mild laboratory‐confirmed influenza that was identified by surveillance. Table 4 provides VE results in different age subgroups. Influenza VE was substantially higher among school‐age than preschool children, with 70.9% vs 34.6% for ILI and 44.0% vs −6.2% for mild influenza infection among those vaccinated within 12 months.

**Table 3:** Influenza vaccine effectiveness (VE) for mild laboratory‐confirmed influenza and influenza‐like illness among all 623 preschool and school‐age children

Outcome	Timing of Vaccination	Vaccinated	Not vaccinated	VE (%)
Mild laboratory‐confirmed influenza by surveillance	Within 3 y	n=333	n=290	17.5 (−39.0‐51.3)
	Positive	26 (7.8)	27 (9.3)	
	Negative	307 (92.2)	263 (90.7)	
	Within 12 mo	n=266	n=357	33.2 (−16.9‐62.2)
	Positive	18 (6.8)	35 (9.8)	
	Negative	248 (93.2)	322 (90.2)	
	13‐24 mo ago	n=256	n=367	6.4 (−58.8‐44.9)
	Positive	21 (8.2)	32 (8.7)	
	Negative	235 (91.8)	335 (91.3)	
	25‐36 mo ago	n=240	n=383	−14.6 (−92.2‐31.5)
	Positive	22 (9.2)	31 (8.1)	
	Negative	218 (90.8)	352 (91.9)	
Influenza‐like illness	Within 3 y	n=333	n=290	49.4 (24.0‐66.9)
	With ILI	39 (11.7)	60 (20.8)	
	No ILI	294 (88.3)	229 (79.2)	
	Within 12 mo	n=266	n=357	50.0 (22.4‐68.4)
	With ILI	29 (10.9)	70 (19.6)	
	No ILI	237 (89.1)	287 (80.4)	
	13‐24 mo ago	n=256	n=367	51.9 (24.5‐70.1)
	With ILI	27 (10.5)	72 (19.6)	
	No ILI	229 (89.5)	295 (80.4)	
	25‐36 mo ago	n=240	n=383	42.1 (10.5‐63.1)
	With ILI	28 (11.7)	71 (18.5)	
	No ILI	212 (88.3)	312 (81.5)	

Results expressed in number (percentage) for influenza outcomes and mean (95% confidence interval) for VE.

**Table 4:** Influenza vaccine effectiveness (VE) in the subgroups of 387 school‐age and 236 preschool children

Outcome	Timing of vaccination	School‐age children			Preschool children		
		Vaccinated	Not vaccinated	VE (%)	Vaccinated	Not vaccinated	VE (%)
Mild laboratory‐confirmed influenza by surveillance	Within 3 y	n=210	n=177	30.4 (−28.6‐62.8)	n=123	n=113	−19.5 (−208.1‐53.1)
	Positive	18 (8.6)	21 (11.9)		9 (7.3)	7 (6.2)	
	Negative	192 (91.4)	156 (88.1)		114 (92.7)	106 (93.8)	
	Within 12 mo	n=166	n=221	44.0 (−10.1‐72.3)	n=100	n=136	−6.2 (−174.8‐58.8)
	Positive	12 (7.2)	27 (12.2)		7 (7.0)	9 (6.6)	
	Negative	154 (92.8)	194 (87.8)		93 (93.0)	127 (93.4)	
	13‐24 mo ago	n=166	n=221	18.5 (−51.9‐56.6)	n=90	n=146	−28.4 (−229.2‐49.1)
	Positive	15 (9.0)	24 (10.9)		7 (7.8)	9 (6.2)	
	Negative	151 (91.0)	197 (89.1)		83 (92.2)	137 (93.8)	
	25‐36 mo ago	n=165	n=222	7.1 (−70.8‐49.6)	n=75	n=161	−73.9(−338.2‐28.5)
	Positive	16 (9.7)	23 (10.4)		7 (9.3)	9 (5.6)	
	Negative	149 (90.3)	199 (89.6)		68 (90.7)	152 (94.4)	
Influenza‐like illness	Within 3 y	n=210	n=177	66.4 (30.3‐84.7)	n=123	n=113	34.3 (−4.8‐59.8)
	With ILI	11 (5.2)	25 (14.1)		28 (22.8)	35 (31.0)	
	No ILI	199 (94.8)	152 (85.9)		95 (77.2)	78 (69.0)	
	Within 12 mo	n=166	n=221	70.9 (31.4‐88.6)	n=100	n=136	34.6 (−6.7‐61.0)
	With ILI	7 (4.2)	29 (13.1)		22 (22.0)	41 (30.1)	
	No ILI	159 (95.8)	192 (86.9)		78 (78.0)	95 (69.9)	
	13‐24 mo ago	n=166	n=221	58.8 (11.4‐81.8)	n=90	n=146	43.9 (4.0‐68.6)
	With ILI	9 (5.4)	27 (12.2)		18 (20.0)	45 (30.8)	
	No ILI	157 (94.6)	194 (87.8)		72 (80.0)	101 (69.2)	
	25‐36 mo ago	n=165	n=222	43.7 (−13.9‐72.9)	n=75	n=161	26.7 (−22.7‐57.1)
	With ILI	11 (6.7)	25 (11.3)		17 (22.7)	46 (28.6)	
	No ILI	154 (93.3)	197 (88.7)		58 (77.3)	115 (71.4)	

Results expressed in number (percentage) for influenza outcomes and mean (95% confidence interval) for VE.

## 4 DISCUSSION

Mild laboratory‐confirmed influenza in children was common during influenza seasons in 2014 and 2015. Influenza vaccine uptake was 36%‐44% among Hong Kong children, and overall influenza VE was moderate (42%‐52%) for ILI. Influenza vaccination was effective against ILI but not mild infection identified by our surveillance. More importantly, influenza VE was substantially higher for both ILI and mild laboratory‐confirmed influenza in school‐age than preschool children.

The prospective cohort design of this study offers an ideal approach for respiratory virus surveillance. A significant proportion of patients with respiratory viral infections only suffer from mild symptoms.^15^, ^16^, ^17^ Patients with influenza may only have mild cough and rhinorrhea. Our investigative approach detected 104 children with ILI and 55 children with mild laboratory‐confirmed influenza during influenza seasons in 2014‐2015. We acknowledge that some ILI cases were not due to influenza because most of these children had not been tested for influenza. Nonetheless, we believe that these numbers would be a better reflection of the clinical spectrum for influenza infections in children.

Randomized controlled trials demonstrated efficacy of influenza vaccination^4^, ^5^, ^18^, ^19^, ^20^, but such findings being obtained in an optimal setting may not be generalizable to the real‐life situation. A systematic review showed low influenza VE (often less than 60%) for subjects risk of severe infection.^21^ The Influenza Monitoring Vaccine Effectiveness in Europe project found 43% VE in 2011/12.^4^ Multiple confounding factors such as difficulty in matching influenza A antigen for the vaccine strain vs the dominant circulating viruses^22^, suboptimal vaccine uptake, and poor infection control practices influenced influenza VE. This prospective surveillance study was designed in this direction with biweekly surveillance for influenza A and B viruses by molecular methods. Thus, our results supported seasonal influenza vaccine to be an effective public health measure to prevent ILI in local children. Subgroup analyses revealed that influenza vaccination might be more effective against both ILI and mild laboratory‐confirmed influenza in school‐age than preschool children (Table 4). Another local study reported age‐specific VE for influenza‐related hospitalization to be higher in children aged 3‐5 years than 6‐17 years (91.4% vs 58.1%) in 2015‐16.^23^ However, both this and our studies were limited by small sample size in each age group which led to wide 95% confidence intervals and significant overlap of the VE estimates. Similar findings were observed in a study of 2368 inpatients in Beijing in the 2014‐2015 season^24^, a Japanese surveillance in 2013‐2014^25^, and two US studies in 2013‐2014 and 2014‐2015 seasons^26^, ^27^. Our finding supported possible impact of extending the influenza vaccination programs to school‐age children, but further studies are needed to address the cost‐effectiveness of this vaccination strategy.

Our findings of four A(H1N1)pdm09 and one A/H3 in 2014 and three A(H1N1)pdm09 and 13 A/H3 in 2015 among the typable viruses were consistent with local surveillance data of A(H1N1)pdm09 dominance from early January 2014 to early March 2014 and A/H3N2 dominance from late December 2014 to early April 2015 (http://www.chp.gov.hk/en/sas6/101/110/106.html). Over 95% of A/H3N2 in 2014/15 season was A/Switzerland/9715293/2013‐like, which was mismatched from A/Texas/50/2012 (H3N2)‐like virus being included in the recommended seasonal vaccine. Our molecular typing assay was designed to detect both of these A/H3N2 strains. All influenza B viruses by our surveillance were detected in 2014, with nine being in B/Yamagata‐lineage and 10 being in B/Victoria‐lineage. Whereas the unpredictability of circulating influenza B was known^28^, our typing results were concordant with local CHP data for B/Yamagata‐lineage dominance in early 2014 and low activity (8.4% of isolates) of influenza B in 2014/2015 winter season.

Our government has included children aged 6 months to 5 years as a priority group in the influenza vaccination subsidy scheme. Nonetheless, a small local study found <20% uptake rate in preschool children.^7^ In this study, 36%‐44% of children received influenza vaccination annually (Table 1). Uptake of influenza vaccination was also low among local healthcare workers in the post‐pandemic era.^29^ Breaking barriers to accept influenza vaccination should be a public health priority in fighting against influenza outbreaks. Our findings of moderate overall influenza VE for ILI in children aged 2‐12 years together with higher VE against both ILI and mild laboratory‐confirmed influenza among the subgroup of school‐age children may represent an additional strategy where the Government Vaccination Program can be expanded to cover children up to 12 years old.

We found that influenza vaccination was effective against ILI but not mild infection identified by 2‐weekly surveillance during influenza seasons, which was surprising given that all the non‐influenza causes of ILI would reduce the observed strength of influenza VE toward the null. One possible explanation relates to study power that we detected many more patients of ILI (n=99) than mild influenza infection (n=57). On the other hand, influenza‐infected children were reported to have increased susceptibility to co‐infection by other respiratory pathogens, notably pneumococcus and staphylococcus, which increased the severity of their RTIs.^30^, ^31^, ^32^ Thus, it is also possible that influenza vaccination prevented ILI by reducing the children with such co‐infections. Vaccinated and unvaccinated patients with influenza infections may exhibit different health care‐seeking behavior. This study adopted the test‐negative design to define influenza VE as it is less susceptible to bias due to misclassification of infection and to confounding by health care‐seeking behavior relative to traditional case‐control or cohort studies.^14^, ^33^

Different studies reported that influenza vaccination lowered subject hospitalization rate by 50%‐66%.^5^, ^19^, ^20^ In our research proposal, we targeted to recruit 560 children with evaluable study outcomes which was based on the assumptions of 40% influenza vaccination coverage, respective influenza attack rates of 13% and 25% among vaccinees and non‐vaccinees, and 30% dropout during the surveillance period. Whereas our cohort of 623 subjects should be sufficient to detect the difference in ILI between vaccinated and non‐vaccinated children, this study might not be sufficiently powered to detect the observed difference in laboratory‐confirmed influenza among our subjects. Another limitation relates to our inability to detect symptomatic moderate‐to‐severe laboratory‐confirmed influenza. Students absent on the days of school visits were not sampled. Because of frequent school visits, our nurses could not arrange home visits for collecting FNPS from children with ILI for influenza testing. For influenza A(H1N1)pdm09 infection, the revised WHO ILI case definition with fever and cough had low sensitivity (36%) but higher positive predictive value (42%) and positive likelihood ratio (13.3) than the other case definitions.^34^ In a Taiwanese study, the presence of fever, cough and sneezing had the best specificity (77%) for laboratory‐confirmed influenza.^35^ Different ILI case definitions adopted in Europe, USA, and Taiwan had comparable accuracy in sensitivity and specificity, and clinical diagnosis of ILI was useful for providing valuable information for surveillance purpose. This study defined ILI by revised criteria that included other respiratory and constitutional symptoms. It is likely that our patients with ILI had influenza during influenza seasons. Another limitation relates to low participation rates of kindergartens (10/238) and primary schools (5/97), which raised concern if our subjects were recruited by convenience sampling. Nonetheless, we believe our surveillance data were generalizable because these kindergartens and primary schools were identified in sequential random batches from all in our target geographic regions that were registered under the Education Bureau. This study was also limited by the lack of surveillance data for subjects’ household and class contacts. Most secondary cases in this surveillance were expected to have minimal influenza symptoms due to low viral load. Unless we collect surveillance samples from subjects’ close contacts, we shall miss these secondary cases by only calling them for any ILI symptom.

In conclusion, mild laboratory‐confirmed influenza was common among children during influenza seasons in 2014‐2015. Moderate overall influenza VE was found for ILI, and subgroup analyses suggested higher VE for both ILI and mild laboratory‐confirmed influenza in school‐age children. Whether vaccination prevented influenza transmission within families or classes remained unanswered due to lack of reported secondary cases.

## CONFLICT OF INTEREST

All authors declared no conflict of interest.
